# Sources of strength: a process evaluation of a university-high school partnership to promote mental health protective factors

**DOI:** 10.3389/fpubh.2024.1426922

**Published:** 2024-08-27

**Authors:** Duke Biber, Gina Brandenburg

**Affiliations:** ^1^Department of Health Sciences, James Madison University, Harrisonburg, VA, United States; ^2^Department of Sport Management, Wellness, and Physical Education, University of West Georgia, Carrollton, GA, United States

**Keywords:** social support, suicide, opioid, adolescent, positive psychology

## Abstract

The purpose of this manuscript was to discuss the implementation process of a student-led positive psychological and behavioral program (i.e., Sources of Strength) at a local high school to prevent opioid use and suicide behavior. Over the course of 2 years of programming, university undergraduate students worked alongside and mentored high school students to implement school-wide and focused campaigns that targeted each of the domains of the Sources of Strength wheel (i.e., mental health, family support, positive friends, mentors, healthy activities, generosity, spirituality, and medical access). The summed total student reach for 2 years of implementation was 8,682 students. The average participation was 456.95 students per campaign. The average percentage of the school population that engaged in each campaign was 34.7%. While no outcome opioid use or suicide behavior data were collected, the participation in the programming was high. Universities can continue to engage with local high schools to provide support, collaboration, and mentorship to promote positive and supportive school culture. Using university undergraduate students to serve as leaders can provide them with applied learning opportunities, mentorship for high school students, and reduce the expectancy for high school staff to establish the program on their own.

## Introduction

Two of major public health challenges that contribute to adolescents’ mortality include opioid drug abuse and suicide behavior. According to the National Vital Statistics Reports (NVSS) from the CDC, suicide ranked among the top 10 leading causes of death in the United States in 2014 [(1), p. 1]. More specifically, 17.4 percent of deaths among the 10–24 age group were caused by suicide [(1), p. 10]. Data from the Youth Risk Behavior Surveillance shows 15.8% of high school students the ages have considered suicide, 7.8% have attempted suicide and 2.4% have necessitated medical care following a suicide attempt (2). There are also sex differences in suicide ideation and attempts. For example, females are twice as likely to attempt suicide than males, but males are more likely to die from suicide when compared to females (males = 9.4/100.000; females = 2.7/100.000) (3). This is since males tend to engage in more lethal means of attempted suicide than females. The rates of suicide are significantly higher among adolescents than any other age group. Between 1999 and 2016, there was a 250% increase of opioid-related deaths in adolescents aged 15–19 years (4). This is important because opioid use is strongly associated with and predictive of suicide ideation, planning, and attempts (5). Furthermore, the COVID-19 pandemic has exacerbated many risk factors for opioid use and suicidality, including family strain, social isolation from friends, lack of structure at school, and overall boredom (6, 7). Many school-level factors also contribute to mental health problems and suicidality, including bullying, unhealthy school climate, unsupportive teachers, and isolation (8). However, positive emotional expression, behaviors, and support have been found to buffer against opioid use and suicide (9, 10).

### A model for positive coping

One model that promotes positivity and flourishing at the individual and group level is the Broaden and Build Model (11). This model posits that positive emotional expression promotes an upward spiral of thought-action repertoires. This means that positive emotions broaden an individual’s perspective of a given situation, counteracts negative emotions, and enhances psychological resilience (12, 13). These benefits, in turn, lead to an upward spiral of thoughts and behaviors in which an individual engages with the world with more curiosity, discovery, knowledge, and skills (12). Simply stated, positive emotions broaden an individual’s perspective of a situation, leading to novel thoughts, activities, and relationships, which results in enhanced personal resources, promoting optimal wellness (14). Negative emotional expression, on the contrary, narrows an individual’s perspective of a situation as a way of ensuring survival from an anthropological perspective. For example, during the hunter-gather period of history, when a hunter stumbled upon a predator like a bear, the negative thought of fear prepared them for one specific action, fight or flight. Most humans do not experience such life-threatening, fear-inducing situations in the twenty-first century. However, the negative emotional response of fear or anxiety during an exam, meeting new friends, or trying out for a team in high school has the narrowing effect today of fight-or-flight, regardless of the situation (13). Negative emotional states, such as stress, anxiety, and depression are all associated with opioid abuse and suicide thoughts, planning, and action in high school (15, 16). Given the association between negative emotions and opioid abuse and suicidality in the high school population, along with the exacerbation by the COVID-19 pandemic, it is so important to implement upstream programming that promotes a positive individual, school, and community culture.

### Sources of strength program

Universal suicide prevention programs, including screening programs, gatekeeper training, mental health literacy training, skills training, and crisis referral can all play a role in whole-school prevention (17). However, primary prevention and actual intervention may require the combination of multiple training strategies, such as school-based programming that utilizes peer-driven awareness and skills training (17, 18). An evidence-based program that promotes high school culture change through positive school-wide campaigns is Sources of Strength. The mission is to prevent suicide, violence, bullying and substance abuse through “training, supporting, and empowering both peer leaders and caring adults to impact their world through the power of connection, hope, help and strength,” (19). Peer and adult leaders help change the perception of typical norms and coping behaviors at the high school by modeling and encouraging their friends to engage with trusted adults to increase communication, enforce the expectancy that friends seek support when needed, and identify and use positive coping mechanisms (20). It is necessary to publish the process of utilizing and training university mentors to come alongside high schools as this is a sustainable model of programming that can benefit both the college mentors and adolescent mentees (21). Further, college students can serve as role models, provide support and encouragement, and can often have more influence than parents or teachers as they are non-authoritative (22).

Sources of Strength focuses on diverse pillars, or campaigns, that protect against suicide behavior and opioid use. This is known as the Sources of Strength Wheel, and consists of mental health, family support, positive friends, mentors, healthy activities, generosity, spirituality, and medical access, each of which can be promoted through school-wide campaigns (23). Rigorous evaluations of Sources of Strength showed that this program can enhance social connectedness to peers and adults at school, promote peer referrals and help-seeking, and decrease maladaptive coping for leaders (20, 24). As expected, these two studies focused primarily on student outcomes, with less detail given to the process of implementation, capacity development, and campaign design. The remainder of this manuscript explains the process of training university undergraduate students to “Adopt-A-High-School” and implement the Sources of Strength program alongside high school peer leaders over the span of 2 years to promote protective factors against opioid use and suicide. The funding and study did not evaluate student-level opioid and suicide risk, but rather the process of implementing mentor-guided protective factor programming through Sources of Strength.

## Methodology

The following sections outline (1) capacity building through the development of the leadership team, (2) campaign design, and (3) implementation of successful programming.

### University undergraduate student leadership

A group of expert faculty members with backgrounds in health and community wellness, health promotion, and health psychology received external funding from the Georgia Department of Behavioral Health and Developmental Disabilities to implement the “College Adopt-A-School” initiative. The funding for the program was aimed to reduce opioid and suicide risk by promoting protective factors through Sources of Strength campaigns, however, no student-level suicide or opioid use data was collected. The university was able to hire undergraduate student assistants to help implement Sources of Strength at the adopted high school in the southeast. The university-high school model was chosen because of previous research supporting the potential benefit to the college student mentors, the individual high school students, and for sustainability of the program (25–27). Over the course of 2 years, 15 undergraduate students were hired to work 10 h per week to conduct a needs assessments of the partner high school, communicate with and mentor the high school peer leader team, collaborate with adult mentors at the high school, create all necessary programming, and implement school-wide and focused programming. The undergraduate students were involved with every aspect of the program, from developing budgets for each campaign, leading meetings with students and staff, and collecting data on program implementation. The undergraduate student leadership team was also trained in the Strategic Prevention Framework. Through a two-day, face-to-face training, students learned the process of assessment, capacity, planning, implementation, and evaluation to ensure sustainability and cultural competence of the Sources of Strength program at the high school (28). Throughout implementation, the undergraduate student leadership team helped train the high school peer leader team in the Strategic Prevention Framework as well. The undergraduate leaders met with the university faculty team once per week to discuss successes, barriers, and ask any questions regarding programming.

### Peer leadership team

A diverse group of high school students volunteered to participate in Sources of Strength training and comprise the peer leadership team for their school. The use of peer leaders is grounded in social learning theory, in which social networks and modelling of behavior can help establish new school-wide norms and health behaviors (20, 29). Per Sources of Strength training protocols, the goal for the peer leadership team is to represent at least 10% of the student body population size. A total of 135 high school peers were trained to help lead the Sources of Strength program, which was 10.8% of the high school population. An additional 13 high school faculty and staff volunteered to sponsor the program over the 2 years. These students worked alongside the university undergraduate leadership team to identify needs at the high school, develop Sources of Strength campaigns, and implement the campaigns at the school. The peer leaders met bi-weekly with the university undergraduate leadership team for 1 h, during which they ate lunch together, played team building games, and developed campaigns to implement. The goal was to segment these meetings into one part fun, one part sharing, and one part planning (23). It is important to note that the peer leadership team led all creative meetings and discussions, helped conduct needs analyses of the school, and were discerned whether each campaign met the culture and needs of the school. The peer leadership team was meant to be the heart, soul, and catalyst for positive culture change at their high school.

### School demographics

The school had a total of 1,250 students during the time of implementation. The racial makeup of the school during time of implementation was 74% White, 11.2% Hispanic, 9.6% Latino, 4.9% biracial, 0.3% Asian, and 0.1% American Indian. In addition, 43% of the student population was free or reduced lunch recipients. However, specific student demographic data per campaign was not collected.

### Campaigns

Over the course of 2 years of programming, university undergraduate students worked alongside and mentored high school students to implement school-wide and focused campaigns that targeted each of the domains of the Sources of Strength wheel. These campaigns were chosen based on the Strategic Prevention Framework and assessment that was conducted by the peer leaders, adult leaders, and university undergraduate leadership team. Further, each campaign fell within one of the key eight evidence-based categories of prevention (i.e., physical health, mental health, family support, positive friends, spirituality, mentors, generosity, and healthy activities) as outlined by Sources of Strength (19). A table of all campaigns implemented can be found in Table 1. During each campaign, two undergraduate students were in charging of measuring reach, in which unique students were counted by both students through a tally sign-in form for high school participants.

**Table 1:** Sources of strength campaign details.

Campaign title	Sources of strength wheel domain	Description	Direct reach	%
Schoolwide Sources of Strength Kickoff	All domains of Sources of Strength Wheel	Sources of Strength interactive mural to promote awareness, encouragement, and support	800 students	64%
Graduating Seniors Celebration	Family Support Positive Friends Mentors	During COVID-19, graduating seniors were provided a mini parade to collect caps, gowns, yard signs, and letters of encouragement as they graduated.	325 students	26%
Summer Graduation Letters of Support	Mental Health Mentors Spirituality	Letters were written to all seniors with mental, emotional, and spiritual encouragement through the first COVID-19 wave.	325 students	26%
Personal Protective Equipment Campaign	Healthy activities Medical Access	Every student received a mask, water bottle, and hand sanitizer to promote safety, reduce anxiety, and inclusion to return to the classroom.	1,250 students 85 classrooms	100%
Strength of Lions Week	All domains of Sources of Strength Wheel	A Sources of Strength hallway with a mascot Lion mural and other SOS campaigns were constructed to engage schoolwide participation.	750 students	60%
Sources of Strength Motto PSA	All domains of Sources of Strength Wheel	High school peer leaders and adult mentors recorded their own “We are Sources of Strength” video to promote inclusion, originality, and ownership.	30 students/staff in video.	2%
			1,250 students viewed	100%
Gratitude Tree	Mental Health Spirituality	Students wrote gratitude letters to high school peers and teachers. They also posted gratitude “leaves” on a “Tree of Gratitude” mural.	462 students	37%
Self-Love and Self-Care	Spirituality Mental Health	Students completed a questionnaire to learn their preferred method of self-care and created positive social support posters throughout the school.	700 students	56%
Key Club Campaign	Positive Friends Mentors Generosity	The Key Club was trained in Sources of Strength to begin spreading the hope, help, and strength message.	20 students	2%
Freshman Honors Celebration	Mental Health Mentors	High GPA/attendance freshmen were celebrated by high school faculty.	50 students	4%
Black History Month	Spirituality Positive Friends	Black history trivia and murals were created to promote inclusion, diversity, and support for minority students from the student body.	192 students	15%
Healthy Activities Week	Healthy Activities	Students engaged in healthy nutrition and yoga.	42 students	3%
Special Olympics Field Day	Positive Friends Mentors Healthy Activities	A Special Olympics with field-day activities for students with disabilities was implemented.	60 students	5%
Graduating Seniors Celebration Year 2	Family Support Positive Friends Mentors	During the second wave of COVID-19, graduating seniors were provided a celebration to collect caps, gowns, yard signs, and letters of encouragement as they graduated.	300 students	24%
Back to School Freshman Camp	All domains of Sources of Strength Wheel	Incoming freshmen were trained in Sources of Strength and provided adult/upperclassmen support as they entered high school.	125 students	10%
My Stereotype/My Truth	All domains of Sources of Strength Wheel	Stereo murals were created for students to write how they were “stereotyped” at school and how they really wanted to be known.	230 students	18%
Faith, Hope, and Courage	Positive Friends Generosity	Murals of Faith, Hope, and Courage allowed students to post sticky notes of what fulfilled each of these categories at school.	249 students	20%
Celebrate Culture	Healthy Activities Spirituality Family Support Positive Friends	Continent murals allowed students from each continent to celebrate their heritage, or students who wanted to travel to that continent to engage in conversation.	272 students	22%
Diversity Week Lesson Plans	All domains of Sources of Strength Wheel	Diversity t-shirts designed by students were distributed along with online lesson plans for teachers to use to promote Sources of Strength.	85 classrooms 1,250 students	100%
Social Media	All domains of Sources of Strength Wheel	University and peer mentors posted on social media to implement Getting the Word Out, Trusted Adults, What Helps Me, We Belong, Thankfulness Challenge, and I Am Stronger campaigns each month.	5,961 impressions 790 users reached	N/A
Total Reach			8,682 students	
Mean Participation			456.95 students	
Reach Percentage (Student Reach/1,250 total students)			34.70%	

### Evaluation

This section provides process evaluative data for the two-year implementation period. Although there were not any student-level outcome data, this study provides insight into program fidelity, program reach, peer to peer and mentor to peer leadership ratios, as well as barriers to implementation.

#### Program fidelity

Fidelity can be defined as the extent to which essential aspects of a program are consistently maintained throughout implementation (30). This study adhered to the required two-day face-to-face Sources of Strength training for all university and peer leaders, as well as a follow-up refresher training. A total of 15 university mentors were trained to help implement campaigns, which is within the 10:1 ratio of peer to adult leaders desired for Sources of Strength. Furthermore, a total of 10.8% of the total high school population served as peer leaders, which is above the recommended 10% as outlined by Sources of Strength training protocols. Thirteen high school teachers were also trained and served as Sources of Strength trusted adults, which was 15% of the teacher staff and above the 10% suggestion. Bi-weekly meetings were also conducted between university mentors and peer leaders, which were peer-leader-driven, which is a focus of the program as well. Lastly, the university and peer leadership team were able to implement 20 different campaigns, during COVID-19, in both face-to-face and social media modalities. The campaign implementation followed the training protocols, with a school-wide kick-off and closing campaign, with various targeted campaigns throughout the implementation period. Following 2 years of successful implementation alongside the university undergraduate leadership team, the goal was to promote program sustainability without external funding. The undergraduate faculty and students assisted the high school staff and peer leaders to plan and develop monthly campaigns to be implemented in year 3, along with the domain of the Sources of Strength Wheel targeted with each campaign.

#### Reach

One of the primary outcome measures of success for implementation was the reach of the Sources of Strength program. This was measured as the total number of individual students who participated, along with the percentage of participation (measured as the number of participants divided by the total number of students at the high school) (see Table 1). The summed total student reach for 2 years of implementation was 8,682 students. The average participation was 456.95 students. The average percentage of the school population that engaged in each campaign was 34.7%. The authors did not track repeat attendees and/or new attendees for each successive campaign. It would have been helpful to understand the number of students who attended every campaign, as well as how many new students attended in each successive campaign.

#### Barriers

There were a variety of barriers to successful implementation of the program as discussed during the university student meetings with program faculty. A list of barriers was noted by a university student scribe each meeting throughout the 2-year implementation period, with faculty assisting to identify solutions. One major barrier was communication with the partner high school. The undergraduate students expressed difficulty communicating with the high school adult mentors (i.e., teachers and staff), and coordinating meeting times with the high school peer leader team. Identifying the best method for communication, such as through the GroupMe messaging application, ensured that all stakeholders understood how to best communicate. Also, identifying the best time for communication was important, in which high school staff interacted mostly in the early mornings or during preparation periods, whereas the peer leaders responded best in the early to late evenings.

Another barrier to implementation was the perception by the peer leaders that they could not be vulnerable and authentic when creating school programming due to teacher supervision and bias. To overcome this, the bi-weekly planning meetings included only the university leadership team and the high school peer leaders. The high school adult mentors had the opportunity to check in on progress, communicate through the GroupMe, or meet with the peer leaders at a different time during the week if they desired. The rationale for involving only university and high school students in the planning meetings was to remove any potential bias, coercion, or pressure that could have come from adult mentors with dual roles as the teachers of these students.

Lastly, the high school student body participated in dozens of sports, extra-curricular activities, and afterschool events. Scheduling Sources of Strength camp gains was often difficult due to the number of school-endorsed events that were already taking place. The university and high school students implemented as many campaigns as possible during the school day to engage the largest reach possible, and not compete with after school events. In terms of facilitating factors that helped implementation, the faculty allowed bi-weekly meetings to be held in their classrooms before, during, or after school. They also allowed classrooms to be used for creation and storage of needed campaign materials. The high school front office staff also supported the program by allowing the use of mass media messaging over the school public address system and on television during the morning announcements. The front office staff also helped with room or field reservations for events, coordinating meetings with school administration, and providing parents with registration forms to be part of the peer leader team. Overall, the program was made possible because of the high school’s buy-in and willingness to help coordinate program implementation, and the ability of the university leadership team to overcome the barriers.

## Discussion

The purpose of this manuscript was to discuss the implementation process of the Sources of Strength program, which aimed to promote protective factors against suicide behavior and opioid use in high school students. Overall, the implementation of the Sources of Strength program at a southeastern high school was successful for a multitude of reasons. First, this study followed the protocol as outlined by Wyman et al. (20) in which there was school and community preparation, adult and peer leader training, and continuous, student-driven schoolwide campaigns. The university collaborated with a high school by giving voice and choice to the high school staff and students to determine the needs to promote positive culture change. Research supports the notion of engaging key stakeholders and community members to implement effective and needed programming (31–34).

The implementation of Sources of Strength was also effective based on peer to student body ratio numbers, high school student population reach, and planned implementation of the program following the end of the funding cycle. First, this study met the 10% peer leader to school population ratio, with a 10.8% ratio over the two-year implementation period. The college mentors promoting inclusion, social connection, and meaning may have influenced high school student desire to participate (21, 35). The current study also had consistent campaign implementation for 2 years, which is much longer than the 4-month outcome evaluation of the program (20). The average campaign reach was 34.7%, and previous randomized-controlled trial of Sources of Strength had an average campaign reach between 59 and 100% (20). The reach of the current study may have been lower (*M* = 34.70) as the campaigns were implemented throughout the COVID-19 pandemic with some staggered school attendance and closures. There are not many Sources of Strength process evaluations to compare this study to in terms of reach for each campaign and university-community partnerships. That said, the reach number is still important to track as a previous study found student-level reach to be a concern of Sources of Strength programming (24).

Lastly, the Sources of Strength program was deemed effective based on fidelity to the training protocol as well as community-driven campaign development and implementation. This is important because a meta-analysis of school-based prevention programming found fidelity to be commonly undocumented and/or unreported, which can greatly impact effectiveness, sustainability, and translation (36). For example, on implementation of Sources of Strength had over 97% fidelity and significant effectiveness (20). It is important to note that school-based programming that is supported by intermediary organizations (i.e., the university setting and leaders in this study) can promote capacity, fidelity, and sustainability (37). Through the support of the intermediary support, such as the university faculty and students, the partner school was potentially able to combat many of the common barriers to fidelity and overall program implementation like competing teacher responsibilities, logistical barriers, and lack of support for programming (38). However, future research should evaluate how fidelity impacts school-level and student-level outcome variables related to mental health and suicidality.

### Limitations

There were limitations to consider throughout the implementation of Sources of Strength. For example, direct outcome measures of suicide behavior, opioid use, social connectivity, or positive emotional states were not collected due to restraints from the funding agency. A recent RCT found Sources of Strength to improve student social connectedness and awareness of help seeking, while also instilling a common language to discuss mental health across students and staff (24). Future implementations should continue to collect baseline and follow-up data to evaluate the effectiveness of each Sources of Strength campaign, or the effectiveness across the entire implementation period. In addition, data was not collected on university student experiences, other than the barriers while implementing the program. Qualitative feedback from university leaders is needed to understand their experiences mentoring and implementing programming. Another limitation is the funding itself. Not every university and high school partner has external funding to implement Sources of Strength. It is important to modify or creatively implement the program so that materials and supplies can be created rather than purchased. From a university perspective, it is also possible to recruit undergraduate volunteers, service-learning students, or internship students in need of high-impact practices, rather than paying student assistants. To further assess program effectiveness, it is necessary to evaluate the experiences of the university undergraduate leaders along with the adult mentors at the high school throughout implementation.

## Conclusion

While no outcome data were included in this manuscript, previous studies indicate that high school students who communicate with trusted peers and adults tend to express healthier coping and an overall trajectory toward a healthier adolescence (39). An analysis of Sources of Strength across 18 high school sites also found that such training improved peer leaders’ adaptive norms, connectedness to adults, peer support, and school engagement (20). Universities should continue to engage with local high schools to provide support, collaboration, and mentorship when desired. Using university undergraduate students to serve as leaders can provide them with applied learning opportunities, mentorship for high school students, and reduce the expectancy for high school staff to establish the program on their own. Emphasis should be placed on both process and outcome evaluations, with a focus on community data-driven decisions, voice, and choice in terms of Sources of Strength programming.

## Data Availability

The original contributions presented in the study are included in the article/supplementary material, further inquiries can be directed to the corresponding author.
